# Evolutionary Shaping of Adult Hippocampal Neurogenesis in Mammals–Cognitive Gain or Developmental Priming of Personality Traits?

**DOI:** 10.3389/fnins.2017.00420

**Published:** 2017-07-21

**Authors:** Hans-Peter Lipp

**Affiliations:** ^1^Institute of Evolutionary Medicine, University of Zurich Zurich, Switzerland; ^2^Institute of Anatomy, University of Zurich Zurich, Switzerland; ^3^Department of Physiology, School of Laboratory Medicine, University of Kwazulu-Natal Durban, South Africa

**Keywords:** adult neurogenesis, natural selection, evolution, hippocampal functions, cognition, comparative, personality, genetic assimilation

Adult hippocampal neurogenesis (AHN) in mammals peaks in early postnatal/juvenile periods and is strongly down-regulated thereafter. Depending on species, it may disappear rapidly in adult individuals, or persist at very low levels for a lifetime. Commonly, higher levels of AHN in mammals are thought to provide mental flexibility allowing for adapting to new ecological niches. But why does natural selection not prevent down-regulation of AHN, and why should a rudimentary proliferation rate in humans provide reproductive fitness even for aged individuals? The problem is compounded by species-specific behavioral manifestations of hippocampal functions that depend on brain size and ecological niches. Moreover, in laboratory rodents, proliferation levels of AHN and behavioral covariates appear unpredictable and context-sensitive. Conversely, one might ask why evolutionary mechanisms tolerate in nearly all mammals a certain level of early postnatal or subadult AHN. Specifically, the hypothesis of cognitive flexibility appears odd in species in which AHN is massively reduced in early infancy such as in humans. I suggest that early but not late AHN plays a hidden role in developing randomly different epigenetic personality traits in local populations. Such traits may counteract or enhance natural selection of the underlying genetic architecture—a process known as genetic assimilation.

In mammals, protracted neurogenesis occurs in subventrical zones (SVZ) from which neuroblasts migrate rostrally to the olfactory bulb (rostral migratory stream, RMS) and from a secondary proliferation zone in the dentate gyrus, the subgranular zone (SGZ). The ongoing postnatal proliferation there is denoted as “adult” hippocampal neurogenesis (AHN). Molecular markers for migration and differentiation are often not correlated with basic levels of AHN in many species. For example, doublecortin (DCX) is a reasonable proxy for estimating proliferation rates in mice and rats. In other species such markers persist for long periods after the cessation of proliferation or appear even generated *de novo* (Amrein, 2015; Penz et al., 2015; Lipp and Bonfanti, 2016). Therefore, AHN and its potential relation to natural selection will refer here to simple proliferation only. After all, it is the dogma-breaking role of persisting neurogenesis that dominates the public view of AHN.

## The main problems

The evolutionary role of AHN in mammals is not understood. The most straightforward explanation shared by many has been offered by Kempermann (2012, 2016) who claims that AHN is an evolutionary recent addition since it is linked with the mammalian hippocampus, which itself is unique for mammals. As the human hippocampus is mediating complex forms of memory, the putative beneficial role of AHN for human memory is extrapolated from rodent studies to humans and to other mammalian species. Thus, cognitive flexibility originating from AHN should provide mammalian species with superior abilities for adapting to new environments. This view bears some problems, namely the role of the hippocampus in mammalian behavior, the different levels of AHN in various species, the differential down-regulation of AHN across species, and the process of natural selection in small populations.

## Does natural selection act on behavioral traits depending on AHN?

To answer this, one would need to know what behaviors and abilities are clearly correlated with hippocampal structural traits including AHN. Perhaps surprisingly, the situation is not clear since even laboratory rodents show a bewildering variety of hippocampus-dependent behaviors, mostly ignored by AHN research. For one, they include natural behaviors such as food burrowing and nest building (Deacon et al., 2002), and social behavior (Ely et al., 1976). Better known are many learning paradigms including simple two-way avoidance (Lipp et al., 1989) and cognitive behaviors such as spatial navigation and pattern separation.

**Figure 1:**
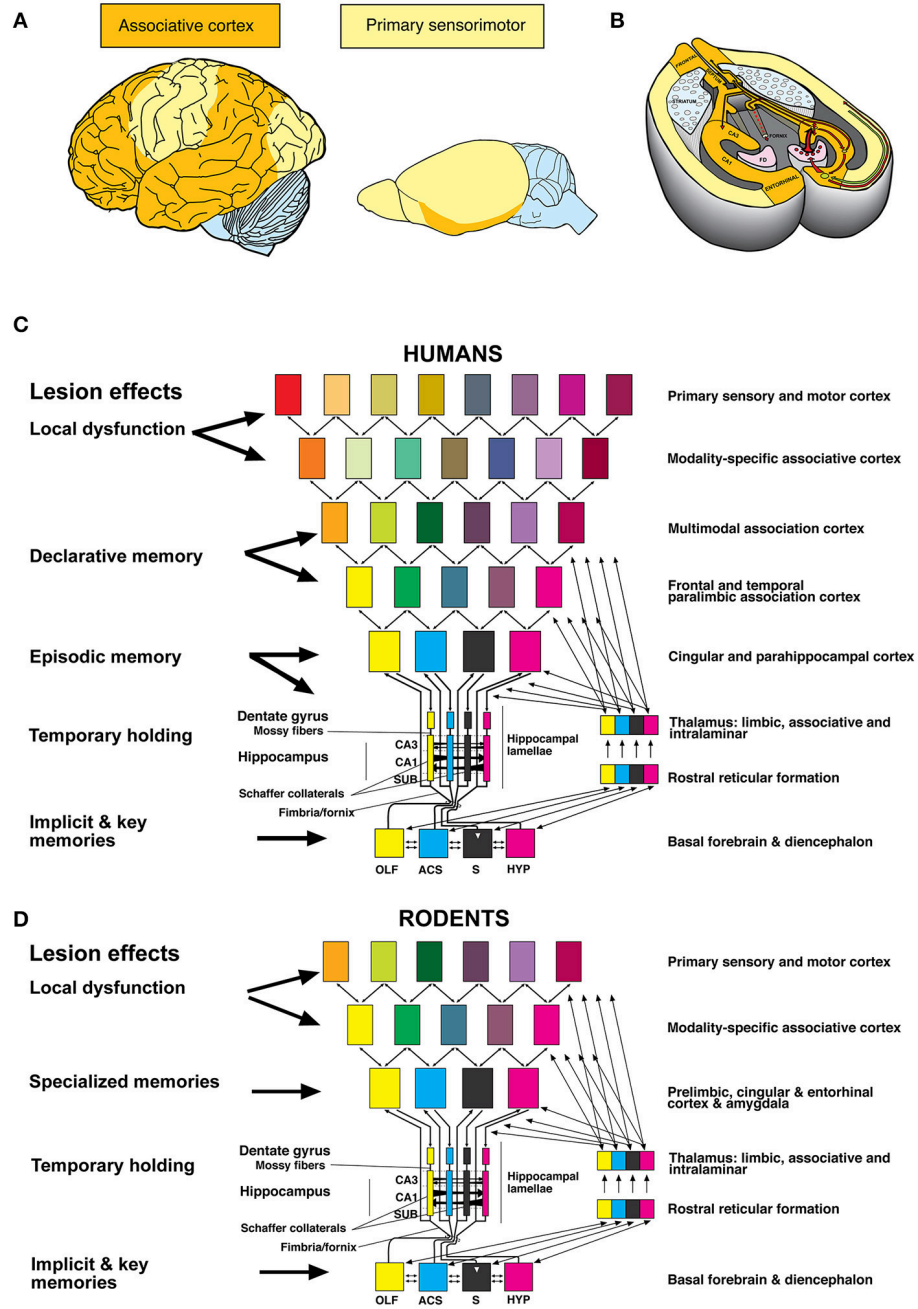
**(A)** Proportions of associative and sensori-motor association cortex differ in humans and rodents, see also (Dong, 2008). **(B)** Associative cortex in rodents is formed mostly by the hippocampal formation. **(C)** Human hippocampal loops receive reduced columnar activity patterns from cortex areas, aligning them to parallel (“trisynaptic”) loops that permit transformation of input/out patterns by Schaffer collaterals in CA3 and CA1. Different colors of rectangles indicate a progressive reduction of cortical activity pattern for representation in the hippocampal formation (Lipp, 2015). **(D)** Corresponding view in rodents in which the input to the hippocampal loop system originates chiefly from non-associative cortex regions but also from subcortical structures. Random dispersal of newly born cells in the dentate gyrus may prime the early postnatal development into different behavioral phenotypes and personalities.

Why is such diversity not known for humans? The human hippocampus interacts chiefly with higher-order association cortex mostly lacking in rodents (Figures 1A,B), see also Dong (2008). In addition, the rodent hippocampus integrates less “cognitive” input with subcortical limbic structures (Figures 1B,D). In both species (Figures 1C,D), the hippocampal loops form the ultimate associative cortex. However, in rodents this structure must also handle functions relegated to specialized cortex regions in humans (Bergmann et al., 2016), being therefore multifunctional. Furthermore, the rodent hippocampus blends hypothalamic and basal forebrain activity into input/output loops connecting to fronto-limbic cortical areas. Thus, the hippocampus is likely to be involved in modulation of many species-typical behaviors. The prevailing uncritical bidirectional extrapolation from human hippocampal functions to rodents and *vice versa* has led to some ill-founded views.

For example, many researchers and editors believe now that AHN is specifically critical for complex tests involving pattern separation, the human ability to memorize fine-grained differences in spatial or contextual environments (Sahay et al., 2011). Yet the behavioral evidence for this conclusion is shaky as it was obtained in mice exposing them to test situations requiring gradually recognizing subtle differences in spatial arrangements of threatening situations. But small rodents cannot afford to discriminate subtleties of threats such as the color and size of a cat and must react immediately. Obviously, AHN-dependent pattern separation is useful for survival of rodents only if it works at once, and this demonstration is still lacking.

## Can variable structural traits in granule cells and mossy fibers mediate behavior and being targeted by natural selection?

The extent of the so-called intra/infrapyramidal mossy fiber projection (IIP-MF) correlates genetically and individually with behavioral traits, not all of them being considered as “hippocampal” (Lipp et al., 1989). Variations of IIP-MF in mice co-vary positively with predictability of ongoing behaviors, and respond rapidly to selective breeding and natural selection (Lipp and Wolfer, 1995, 2002, 2013). Therefore, developmental variations in the distribution of the granule cell axons can predict behavioral traits of adults.

On the other hand, it is difficult to find in rodents consistent correlations between individual numbers of newly generated neurons and individual behaviors. A likely reason is that the extent of the IIP-MF is determined during the first postnatal days of rodent pups, and remains rather stable during adulthood. Conversely, AHN in rodents is extremely sensitive to environmental changes, activity and stress levels, and appears influenced by about 190 genes (Kempermann et al., 2006). It also changes strongly during life history as explained below. Thus, functional relations between AHN and behaviors in mice and rats are masked by excessive temporal variability of the structural substrate, possible interactions with learning, and by the multi-functionality of the rodent hippocampus. This may explain the huge number of behavioral AHN studies with discordant results (Lipp and Bonfanti, 2016). Exceptions with more predictable outcome are some naturalistic behaviors. Genetic suppression of AHN resulted in impairments of species-typical behaviors and sucrose preference in mice (Jedynak et al., 2012) and rats (Snyder et al., 2016). Studies of individual correlations showed positive correlations of AHN levels with sucrose preference (Hu et al., 2016), and with roaming in large enclosures (Freund et al., 2013). Conversely, reactions to novelty correlated negatively with the number of proliferating neurons (van Dijk et al., 2016). But even for species-typical behaviors, their relation to AHN appears unpredictable. Yet natural selection requires that behavioral phenotype and genotype should be strongly linked. It is thus difficult to see how natural selection could act on cognitive or species-typical behaviors linked unpredictably to AHN, and even more difficult to imagine selective pressure on increased AHN in species in which it is already sparse early in life.

## AHN is strongly down-regulated but is bottoming out after two years, independent of species

AHN diminishes with age, initially considered as a normal aging process. However, studies in mice showed that AHN is exponentially decreasing after 7 weeks of age till about 4 months—during peak conditions for reproduction—and leveling off afterwards (Ben Abdallah et al., 2010). A strong decline during the first year of life has now been documented repeatedly for humans (Knoth et al., 2010; Dennis et al., 2016, 2017). Weissleder et al. (2016) have reported a further decrease from years 21 till 91, yet starting from a low level. Other markers of proliferative activity in the human hippocampus did not show an age-dependent decline. Thus it makes sense to distinguish early vs. late AHN.

The belief in a functional role of AHN in aged humans is supported solely by a widely cited study by Spalding et al. (2013) even though it remains controversial among experts. Using the decay of radioactive C14 in human hippocampi, they calculated daily turnover rates of 700 cells per day among 20 millions granule cells. However, they miscalculated proliferation levels in mice and concluded—widely cited also—that AHN in 40 year-old humans is comparable in proliferation levels to correspondingly aged mice (for details see Lipp and Bonfanti, 2016).

In comparative terms, the work of Amrein et al. (2011) has shown that strong down-regulation of AHN occurs in many species regardless of life span and ecological conditions. A bottom in proliferative activity is reached in both mice and men after about 2 years, and AHN is continuing at minimal rates independent of the life span of the species. These findings make cross-species comparability of AHN and life history questionable.

For example, we had hypothesized that the decay of AHN in humans might follow a much slower pace, bottoming out at the age of about 30 years (Amrein and Lipp, 2009). This view might have explained some typical transitions in human life history such as the emergence of long-term memory at the expense of short-term memory in children (Yim et al., 2013; Akers et al., 2014). Likewise, it would have fitted the transition from exuberant and reckless juvenile behavior to cautious adult behavior—a characteristic of the maturation of the hippocampus as postulated by Altman et al. (1973). The general idea was that the input-output patterns of the hippocampal formation between the loops crossing the hippocampal formation (Figures 1C,D) would initially be kept malleable by the AHN-dependent production of juvenile excitable granule cells. The down-regulation of AHN would then entail a fixation of input-output relations associated with acquired and species-typical behaviors optimal for a given environment. This concept would make sense in species in which the initial reduction of AHN covers pre- and post-puberty and early adulthood such as in mice or rats. In these species, the altered hippocampal physiology might be of evolutionary adaptive value. Therefore, natural selection might favor strong early AHN followed by a period of fixing behavioral traits. However, in humans the decay period falls into a time window with reduced behavioral expression and cognitive abilities. Moreover, it is accompanied by an enormous (non-proliferative) growth of dendrites and connections in the forebrain. This raises the question whether early peaking of AHN and down-regulation with late bottoming-out is useful for humans at all.

## Why is late human AHN not enhanced by natural selection?

The most parsimonious answer is that continuing low-level AHN does not matter at all, assuming that the addition of 700 new cells per day (if this can be proved) has a negligible impact on adult on human hippocampal functions. Nowadays, this view has become almost heretical. But one might rightly ask why a putative beneficial structural trait has been curtailed in humans and not increased by natural selection. At present there is no answer. A conceptually related question is why natural selection has (regrettably) not increased the intelligence quotient in humans from an average of 100 to one of 140 points. Obviously, reproductive fitness—a benchmark of evolution—is not strongly correlated with cognitive abilities. Its reproductive advantage is counteracted by competing traits such as physical attractiveness and, among males, physical strength and aggression, not infrequently at the disadvantage of courting academics. For human AHN, the reasons are less obvious. Possibly, the emergence of highly excitable young neurons may interfere with the orchestrated development of other granule cell functions (Drew et al., 2016), or it is even impairing normal cognitive function (Walton et al., 2012). Alternatively, hippocampal functionality and granule cell excitability is regulated by other mechanisms than simple proliferation. Whatever reasons, it would seem that AHN in humans is suppressed early in ontogeny, in parallel with neurogenesis in the SVZ, probably also in other primate species (Lipp and Bonfanti, 2016). It is thus difficult to see why natural selection should preserve a small and dwindling cell population in the human dentate gyrus. This would require showing that late human AHN has a clearly beneficial effect on reproductive fitness or is advantageous for the survival of the group members.

## But why is early postnatal AHN maintained even in humans?

On the other hand, comparative analysis across mammals (Amrein, 2015; Patzke et al., 2015) suggests that an initial level of AHN is maintained in most species, at least in those in which age-dependency could be investigated. Differences between orders and species emerge primarily in the time course of the decay within a span of 2 years, and in the molecular differentiation of the newly generated cells (Amrein, 2015). Up to now, a coherent picture of how species differences in AHN relate to ecological conditions must remain speculative, chiefly because of low sample sizes and problems in quantifying age levels. However, the typical time course of AHN in mammals must provide some variable advantages in terms of natural selection, perhaps more in short-living rodents than in primates and other species.

## Early hippocampal neurogenesis may prime randomly the development of personality traits

The problem in humans is to find a useful function for early postnatal neurogenesis during a period of reduced cognition. This function ought to provide a target for natural selection in adults in order to maintain this developmental trait. One might argue that hippocampal neurogenesis in such an early postnatal stage could help in organizing the development of forebrain circuitry underlying cognitive processing, e.g., the emergence of language. But this should have consequences for adult behavior many years later. Yet it remains questionable whether individual differences in language development do have an impact on evolutionary relevant adult behavior.

On the other hand, personality traits such as risk-taking behavior or food preferences must have been obvious targets for rapid natural selection during human evolution, specifically in small populations. In larger populations, genetic variation balances competing traits, permitting rapid natural selection of carriers fitting into a changed environment. However, in smaller groups natural selection, for example for timidity, might rapidly eliminate genetic variation supporting risk-taking behavior. This can reduce the ability of genetically adapting to new environments suddenly favoring risk-takers.

Loss of evolutionary plasticity due to natural selection is a common problem for small populations in many species. A possible mechanism counteracting such processes is the early development of personality traits in absence of genetic variation. The unpredictable occurrence of correlations between AHN and personality traits in isogenic rodents suggests that such personality traits emerge randomly. For example, sucrose preference shows strong genetic variability in mice and humans (Reed et al., 1997). Yet testing inbred mice for sucrose preference shows often a minority of individuals with initial taste neophobia, indicating that this long-lasting trait can develop purely epigenetically, remaining correlated in rats with individual levels of AHN (Hu et al., 2016). Sweet preference is an adaptive trait that seemingly deserves to be naturally selected, fixing it rapidly genetically. However, this might be fatal for a population as soon as sucrose becomes associated with toxins. But if there are epigenetic traits for taste neophobia, these individuals will not be eliminated rapidly and natural selection might start selecting alleles promoting taste neophobia, a process known as genetic assimilation (Renn and Schumer, 2013).

This idea is in line with recent findings of an elegant study using a chemogenetic approach to inactivate selectively perinatally and postnatally born olfactory neurons in mice (Muthusamy et al., 2017). They found that perinatally born neurons were controlling innate fear responses to predator odor, while neurons born 6 weeks later appeared to control the acquisition of novel appetitive odors. Thus, newly added neurons may prime novel preferences without erasing critical responses for survival. A second example is the individual propensity for roaming, often taken as a measure for risk-taking behavior. Again, individual correlations between levels of AHN and “exploratory” activities have been reported for inbred mice (Freund et al., 2013; van Dijk et al., 2016). Therefore, developmental randomization of behavioral traits by means of AHN at very early ages might predict later gustatory and olfactory preferences, plus roaming activity, not only in mice but also in humans. After all, from 110,000 years ago humans had to survive evolutionary critical bottlenecks by switching to a mussel diet (Marean, 2016)—certainly not the preferred food of most primates. Likewise, the presence of roamers and non-roamers in a population helps to find new habitats. Thus, the evolutionary gain of AHN in most mammalian species including humans might not be cognition but regulating the degree of genetic assimilation of behavioral traits of fundamental importance for adaptation to new habitats.

## Author contributions

The author confirms being the sole contributor of this work and approved it for publication.

### Conflict of interest statement

The author declares that the research was conducted in the absence of any commercial or financial relationships that could be construed as a potential conflict of interest.
